# Women's Preferences Regarding Infant or Maternal Antiretroviral Prophylaxis for Prevention of Mother-To-Child Transmission of HIV during Breastfeeding and Their Views on Option B+ in Dar es Salaam, Tanzania

**DOI:** 10.1371/journal.pone.0085310

**Published:** 2014-01-22

**Authors:** Matilda Ngarina, Edith A. M. Tarimo, Helga Naburi, Charles Kilewo, Mary Mwanyika-Sando, Guerino Chalamilla, Gunnel Biberfeld, Anna Mia Ekstrom

**Affiliations:** 1 Department of Obstetrics and Gynaecology, Muhimbili National Hospital, Dar es Salaam, Tanzania; 2 Swedish Institute for Communicable Disease Control, Solna, Sweden and Department of Microbiology, Tumor and Cell Biology, Karolinska Institutet, Stockholm, Sweden; 3 Department of Nursing Management, Muhimbili University of Health and Allied Sciences, Dar es Salaam, Tanzania; 4 Department of Pediatrics, Muhimbili University of Health and Allied Sciences, Dar es Salaam, Tanzania; 5 Department of Public Health Sciences, Karolinska Institutet and Department of Infectious Diseases, Karolinska University Hospital, Stockholm, Sweden; 6 Department of Obstetrics and Gynaecology, Muhimbili University of Health and Allied Sciences, Dar es Salaam, Tanzania; 7 Management and Development for Health, Dar es Salaam, Tanzania; UCL Institute of Child Health, University College London, United Kingdom

## Abstract

**Background:**

The WHO 2010 guidelines for prevention of mother-to-child transmission (PMTCT) of HIV recommended prophylactic antiretroviral treatment (ART) either for infants (Option A) or mothers (Option B) during breastfeeding for pregnant women with a CD4 count of >350 cell/µL in low-income countries. In 2012, WHO proposed that all HIV-infected pregnant women should receive triple ART for life (B+) irrespective of CD4 count. Tanzania has recently switched from Option A to B+, with a few centers practicing B. However, more information on the real-life feasibility of these options is needed. This qualitative study explored women's preferences for Option A vs B and their views on Option B+ in Dar es Salaam, Tanzania.

**Methods:**

We conducted four focus group discussions with a total of 27 pregnant women with unknown HIV status, attending reproductive and child health clinics, and 31 in-depth interviews among HIV-infected pregnant and post-delivery women, 17 of whom were also asked about B+.

**Results:**

Most participants were in favor of Option B compared to A. The main reasons for choosing Option B were: HIV-associated stigma, fear of drug side-effects on infants and difficult logistics for postnatal drug adherence. Some of the women asked about B+ favored it as they agreed that they would eventually need ART for their own survival. Some were against B+ anticipating loss of motivation after protecting the child, fearing drug side-effects and not feeling ready to embark on lifelong medication. Some were undecided.

**Conclusion:**

Option B was preferred. Since Tanzania has recently adopted Option B+, women with CD4 counts of >350 cell/µL should be counseled about the possibility to “opt-out” from ART after cessation of breastfeeding. Drug safety and benefits, economic concerns and available resources for laboratory monitoring and evaluation should be addressed during B+ implementation to enhance long-term feasibility and effectiveness.

## Introduction

The current global plan is to have an AIDS-free generation by eliminating new HIV infections among children by 2015 and keeping their mothers alive [1]. This is to be achieved by reducing the number of new HIV infections in children by 90% and HIV-related maternal deaths by 50% [1].

Despite the wide coverage and acceptance of programs to prevent mother-to-child transmission (PMTCT) of HIV in sub-Saharan Africa, about 90% of children who acquire HIV and the majority of AIDS-related pediatric deaths still occur in this region [2]. In Tanzania alone, where more than 90% of health facilities provide PMTCT services, it was estimated that about 230,000 children below the age of 15 years were living with HIV in 2012 [2], [3] with around 43,000 new pediatric infections per year [4]. Since the majority of children acquire HIV infection from their mothers (90%), the alarming high rates of HIV among women, high fertility rates and less effective PMTCT regimens threaten the goal of achieving the global plan for an AIDS-free generation. Women now make up the majority (760,000) of the 1.4 million people living with HIV infection in Tanzania [2], [3]. The fertility rate in Tanzania is among the highest in the world, (5.7), with an estimated 119,000 HIV-positive women giving birth every year [4]. The HIV prevalence among women of reproductive age (15–49) in Tanzania was around 6.2% in 2011–2012 [5].

Studies in sub-Saharan Africa have shown a reduction of MTCT rates of HIV to 5% or less at 6 months after delivery when prophylactic ART was used during the latter half of the pregnancy and during breastfeeding [6]–[12]. Both maternal and infant postnatal antiretroviral prophylaxis has been shown to reduce postpartum transmission in breastfeeding populations [6]–[16]. Infant formula is challenging due to the prohibitive costs, the stigma associated with non-breastfeeding and inadequate access to safe water resulting in increased infant morbidity and mortality [13], [17], [18]. In addition, the common belief that breast-milk alone does not satisfy the baby makes exclusive breastfeeding a stigmatizing sign of HIV-infection. Furthermore, the mixing of breast-milk and unsafe fluids increases MTCT due to intestinal infections in the infants [19]. Therefore PMTCT through prophylactic ART is a cost-effective strategy to prevent HIV in breastfeeding populations [20].

The World Health Organization (WHO) estimates that pregnant women with CD4 counts of ≤350 cells/mm^3^ account for about 40% of all HIV-positive pregnant women, causing more than 80% of postpartum transmission and 85% of maternal deaths within 2 years of delivery [21]. As of 2010, only 50% of pregnant women living with HIV in sub-Saharan Africa received effective ART for PMTCT [22]. Given the strong evidence of reduced MTCT and improved clinical outcomes if life-long ART was initiated earlier, WHO published new PMTCT recommendations for low-income countries in 2010 proposing earlier ART initiation and continuous, lifelong ART starting at a CD4 count of <350 cells/µL. The recommendations entailed two new PMTCT options for mothers with a CD4 count of >350 cells/µL. Option A was to start prophylactic ARV as early as at 14 weeks of gestation although post-delivery ARV prophylaxis should be given only to the infant until after breastfeeding has ended. Option B was to give mothers triple ART as early as at 14 weeks of gestation, throughout pregnancy and breastfeeding [23]. In 2012, WHO proposed that all HIV-infected pregnant women, irrespective of CD4 cell count, should start on lifelong ART (Option B+) [24]. The choice for a preferred option is to be made at country level depending on feasibility, acceptability, safety, cost and capacity. Tanzania is shifting from Option A to Option B+ [4] but the acceptability, feasibility and sustainability of this endeavor is not known.

The WHO/UNAIDS have urged countries to use qualitative methods to understand women's perceptions, attitudes and preference with regards to Options A, B and B+ in their own local cultural context [25]. This qualitative study aims to contribute evidence on how to best implement the various PMTCT guidelines by providing a client's perspective on preferences, potential uptake and adherence. The information acquired is also highly relevant for strengthening the performance of under-resourced health systems, optimizing real-life effectiveness of PMTCT and saving lives.

## Methods

### Ethical statement

Ethical clearance was sought and obtained from the Institutional Review Board (IRB) of the Muhimbili University of Health and Allied Sciences (MUHAS). We were aware that the interview could harm the women psychologically and/or expose them to the risk of unwanted disclosure of HIV status with potentially serious consequences. Thus, great precaution was taken not to ask very sensitive questions, stop interviewing when the informant was uncomfortable about giving more information and to make sure that the interview was conducted at a place where the informant felt safe and relaxed. Women were assured of the confidentiality of their HIV status within the research team. The interviews were also carried out at confidential places where no one could hear what was being discussed. We did not want to know or record anything that would identify them such as the name of the interviewee, their children, or spouse, nor is their place of residence revealed anywhere in the paper. The identity numbers given during analysis do not follow the order of the interviews. Participation was entirely voluntary and the women were informed that at any time during the IDI or FGD they could decide to opt out at no cost and with no negative consequences whatsoever with regard to her own care. Each woman read and signed the consent form individually (both in the FGDs and IDI) except for one woman who could not read and write who therefore consented through a finger print after the researcher had read the consent form to her.

### Study design

This was an explorative qualitative study using focus group discussions (FGDs) and in-depth interviews (IDIs) in Dar es Salaam, Tanzania between July 2012 and June 2013.

The FGDs were conducted at reproductive and child health (RCH) clinics among consenting pregnant women with unknown HIV status on their first visit. They were interviewed after going through health education sessions and HIV screening but before going for HIV results. In this particular study, FGDs were used because they are typically designed to elicit normative views and perceptions [26]. FGDs also preserve group norms, richness of information, and groups' perspectives in terms of collective judgments [27].

IDIs were conducted among HIV-positive pregnant women already assigned to either Option A or B. These interviews were also conducted among postnatal women on both options before and after the child had their first HIV test at 6 weeks. In-depth individual interviews are thought to be the most appropriate method in this context where HIV infection still carries a lot of stigma and most would not be comfortable discussing such issues in groups. “The one-to-one interview offers both the sense of privacy and the face-to-face opportunity to build the rapport that is necessary for participants to feel comfortable discussing extremely personal topics with a researcher they just met [28]”. Such interviews also allow for rich and vivid answers [28].

### Study site and study population

This study was conducted in Dar es Salaam, the largest commercial city in Tanzania. Dar es Salaam has 3 districts with a total of 213 RCH facilities providing PMTCT services. Of these facilities, 185 are supported by a local organization – Management Development for Health (MDH) – and the remaining sites are supported by Pastoral Activities and Services for People with AIDS in Dar es Salaam Archdiocese (PASADA). Of these 185 RCH facilities in Dar es Salaam, 152 facilities are in the Ilala and Kinondoni districts where this study was conducted.

We conducted a total of 4 FGDs (2 among primigravida and 2 among multigravida) and 31 IDIs. We performed 10 IDIs with women using Option A, one of whom was also asked to give her views on Option B+ and 21 IDIs with women using Option B, 17 of whom were asked to give their views on Option B+. The question about option B+ was mostly asked to women under Option B, given that they had some specific experience of taking medication themselves, most of them without being symptomatic or having low CD4 counts. We reached “saturation of information” already after 10 interviews, but added 7 more to strengthen the information we had collected.

Not all women in Option B were asked about B+ as some had infants who needed their attention and hence were not ready to prolong the interview while some others wanted to end the discussion when it was their turn to get service at the clinic. Most of these clinics were congested with very few staff so women would wait for long before they got service and would want to leave the clinic immediately after being served. The information received from these interviews was considered thick, rich and adequate. Thus, we reached a saturation point where we felt that adding more interviews would not bring forth any new information [29].

### Sampling process and data collection

Adoption of the WHO 2010 PMTCT guidelines started in Dar es Salaam (Ilala and Kinondoni districts) in February 2012 with the implementation of Option A. However, some sites were requested to implement Option B from July 2012 for research purposes. Sixty RCH clinics were assigned to practice Option A and 92 clinics were assigned to practice Option B. The FGDs and IDIs for women in Option A were conducted in July and August 2012 and IDIs for women in Option B were conducted in January and June 2013 as we had to wait for women to deliver and have their children tested by HIV-PCR.

Data collection started after obtaining ethical clearance from the MUHAS Research and Publication Committee and permission from the heads of the respective health facilities. Interviews were conducted in 6 purposefully selected clinics (3 from each district), i.e. clinics were preferred that had enough clients (at least a minimum of 10 new cases per day), confidential rooms or places with enough privacy and located close to public transport to facilitate the homeward journey of the key informant afterwards.

Women were allowed to follow the normal RCH routine care. On arrival at the clinic, nurses give women health education on pregnancy-related issues comprising PMTCT. Just after the health education session and HIV screening for first time attendees, the main author (MN) and one of the co-researchers (ET) identified and approached women eligible for FGDs. They were informed about the research before being asked if they would like to participate. Consenting women were interviewed and tape-recorded. A convenient place was identified by the researchers and agreed upon by the women. Two FGDs were done at one site on 2 different days and 2 others were done at 2 other different sites. One FGD was done per site per day and lasted for 1 hour. The number of participants ranged from 4 to 8, identified only by numbers during the discussion for confidentiality reasons.

The FGDs were divided into primigravidae vs multigravida since we assumed that the experience of previous pregnancies, child bearing and child-care could impact on women's PMTCT preference. Other sub-groups considered include age, occupation and education but the practicalities of getting enough numbers of women of the desired characteristic to consent for FGD all at one time during a particular day at the ANC clinic was challenging as we did not want to interfere with women's or the clinic's daily routine work. We also realized that majority of the women who were ready to talk to us had primary education and had small income business or were unemployed. Most of the well-to-do women go to private hospitals where they attend by appointments and it may be very challenging to hold them up for interviews. However, when examining women's views in the in-depth interviews, these did not differ with age, education or occupation. However, we noted that the multigravida could relate better to the challenges of child-care and medication than the primigravida could do.

IDIs were also conducted among consenting HIV-positive pregnant and delivered women who had already practiced one of the options. Women were purposefully selected so that the sample included women with different characteristics (pregnant, primiparous, multiparous, post-delivery but before screening the child and post-delivery after screening the child) practicing either of the options. These interviews were tape-recorded and they lasted between 10 and 45 minutes. Some of the interviews were shortened due to interruptions from crying babies and in the event of a respondent being unwilling to provide or uncomfortable about providing more information. Table 1 illustrates how key informants were selected for both FGDs and IDIs. More women in Option B were interviewed as they were thought to be a more appropriate group to be asked about their views on Option B+ as they were on triple ART postnatally.

**Table 1:** Data collection model – selection of key informants.

FGD (first visit with no known HIV status)				
ILALA DISTRICT		KINONDONI DISTRICT		
1 FGD WITH 4 PRIMGRAVIDS (Buguruni)		1 FGD WITH 7 PRIMGRAVIDS (Kambangwa)		
1 FGD WITH 8 MULTIGRAVIDS (Mnazi mmoja)		1 FGD WITH 8 MULTIGRAVIDS (Kambangwa )		
**IDIs (HIV positive women – pregnant/post delivery/PCR – assigned to either option A or B**				
ILALA DISTRICT		KINONDONI DISTRICT		
OPTION A	OPTION B	OPTION A	OPTION B	
3 Pregnant women	6 Pregnant women	2 Pregnant women	5 Pregnant women	
	1 Delivered woman (PCR not yet done)	1 Delivered woman (PCR not yet done)	1 Delivered woman (PCR not yet done)	
2 delivered women (baby has had PCR)	2 delivered women (baby has had PCR)	2 delivered women (baby has had PCR)	5 delivered women (baby has had PCR)	

The main author, who is a clinically active obstetrician and gynaecologist in Dar es Salaam, and the second author, a public health specialist, are both female, Tanzanian, familiar with qualitative research methods and fluent in both Swahili and English. They interviewed the women at their respective RCH clinics. Interviews were conducted in Kiswahili (national language) using an interview guide and were tape-recorded. The guide was amended after the first IDI and FGDs to accommodate interesting and valuable thoughts in the next interviews. It was also modified to accommodate a new question on women's views of Option B+ since Tanzania had begun to discuss adopting this policy when the study was ongoing.

A semi-structured interview guide was used in both the FGDs and IDIs. Themes discussed included awareness of MTCT of HIV and knowledge about how to prevent transmission, but the main focus was on issues and reasons regarding the most preferred option (A, B or B+) for PMTCT among breastfeeding women in this setting. The main and second authors alternatively interviewed women or observed and took notes during the FGDs. The IDIs were conducted by either the main or the second author.

### Data analysis

**Figure 1:**
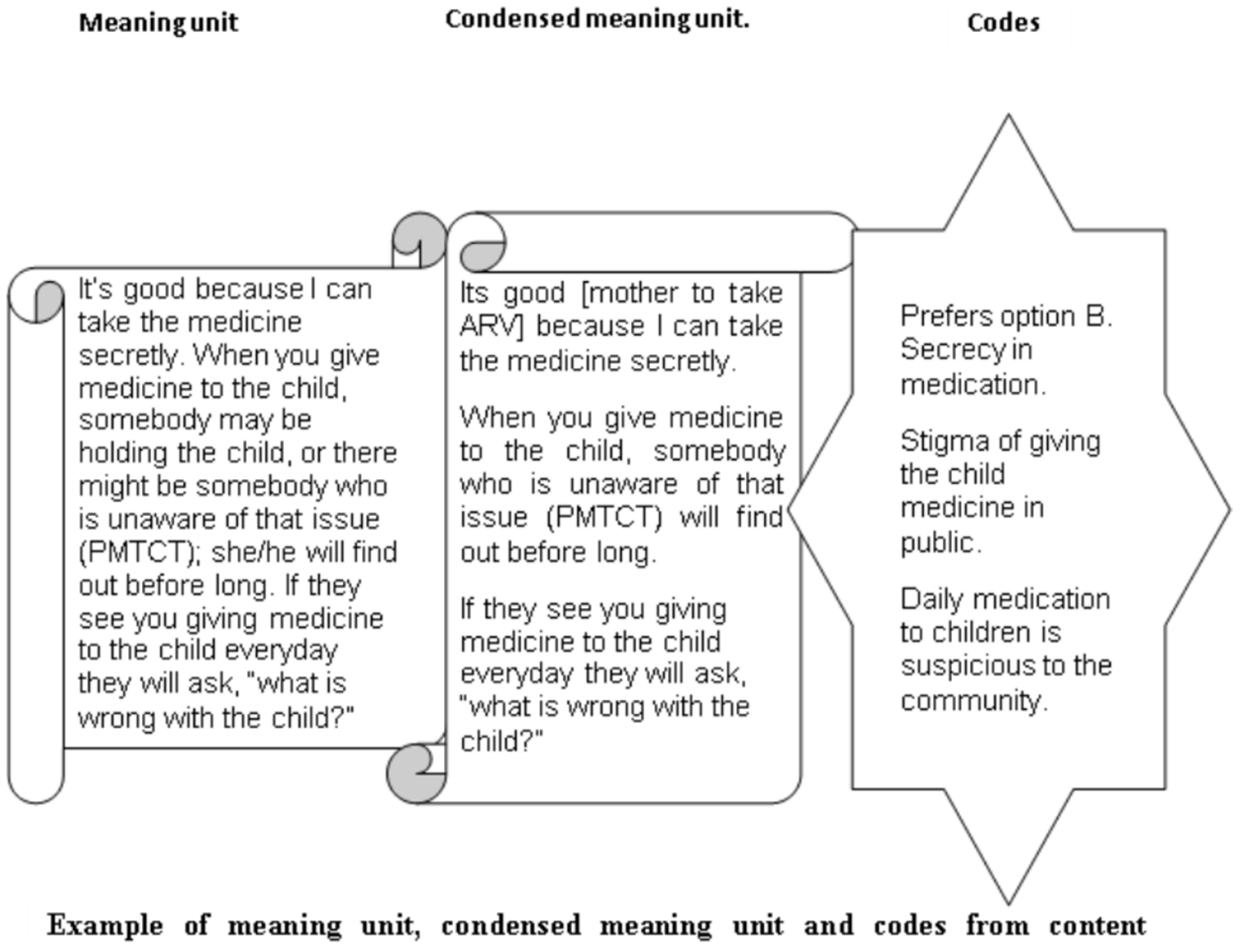
Example of meaning unit, condensed meaning unit and codes from content.

Data analysis began during data collection and evolved throughout the data collection and analysis period as guided by Kvale [30]. The tape-recorded interviews were transcribed verbatim to obtain Swahili transcripts that were later translated into English. Our data consisted largely of interview transcripts, focus group transcripts, memos and field notes. The main author (MN) reviewed the transcripts and compared them to the audiotapes to make sure no part of the interview was missed. We used content analysis to analyze the transcripts. A text unit was selected and then condensed into meaning units and codes (Figure 1) according to Graneheim and Lundman [31]. The codes were thereafter manually sorted into categories and the main theme(s) emerged. MN and ET both independently coded the transcribed data. They thereafter compared their coding for consistency and consensus on data interpretation. The transcripts were also read and codes were reviewed and tested by GB and AME to verify the categories.

### Trustworthiness of the study

“Trustworthiness is the ability of the methodology to capture the reality of those being studied. We believe the results of this study are trustworthy for several reasons: women's accounts were largely in agreement with each other even though the women did not know one another; data saturation was reached before all the interviews were carried out; although the women's accounts go beyond results of previous studies they do not contradict any previous research and are in line with what we found in our previous PMTCT postnatal study [32] and what we know about life in poverty. Furthermore, the first author MN also works as a clinician (gynaecologist and obstetrician) in the same setting and is well acquainted with women's normal attitudes and behaviors towards the challenges of PMTCT in this environment. The interviews were also conducted in the women's native language, Kiswahili. The trustworthiness of the data analysis was further ensured by peer debriefing sessions (mainly MN, ET, HN and CK), using tape recorded interviews and by joint analysis of the data by a research team composed of multiple professions (physicians, experts in public health, professors in public health and clinical immunology).

## Results

### Description of the informants

There were 4 FGDs with a total of 27 women and 31 IDIs. The primigravida who participated in the FGDs were all 18–25 years old; the majority had completed primary education (seven years) were married and were housewives. The multigravida who participated in the FGDs had an age range of 22–37 years; the majority had completed primary education, cohabiting and had small-scale businesses. The age of those participating in the IDIs ranged between 21 and 41 years; the majority were married, had completed primary education, had small-scale businesses, and had disclosed their status to their partners but did not know the HIV status of their partners (Table 2).

**Table 2:** Characteristics of participants in in-depth interviews.

Id no	Age yrs	Marital status	Education	Occupation	Disclosure to partner	Partner's status	Delivered (D) pregnant (P)	Duration on ARV (Mother) weeks	Duration on ARV (Child) weeks	PCR
**1a**	33	Mar	Primary	H/wife	No	Unknown	P	2	-	-
**2a**	34	Mar	Secondary	Business	Yes	Unknown	P	1	-	-
**3a**	27	Mar	College	Agriculture	No	Unknown	P	12	-	-
**4a**	34	Mar	Primary	Business	Yes	Unknown	P	8	-	-
**5a**	38	Mar	Primary	Business	Yes	Positive	D	-	3	-
**6a/b+**	28	Mar	Secondary	Business	Yes	Negative	D	-	12	Neg
**7a**	23	Div	Secondary	H/wife	Yes	Negative	D	-	6	-
**8a**	33	Mar	Primary	H/wife	Yes	Unknown	D	-	24	Neg
**13a**	30	Mar	Primary	Transporter	Yes	Unknown	D	-	28	Neg
**14a**	34	Mar	Primary	H/wife	Yes	Positive	P	4	-	-
**15b**	21	Mar	Primary	H/wife	Yes	Positive	D	-	6	-
**16b**	41	Mar	Primary	H/wife	Yes	Negative	D	-	18	Neg
**17b+**	28	Single	Primary	Business	Yes	Unknown	P	12	-	-
**18b**	38	Single	Primary	Business	Yes	Unknown	P	12	-	-
**19b**	32	Mar	Primary	H/wife	Yes	Unknown	D	-	8	Neg
**20b**	32	Mar	Primary	H/wife	Yes	Unknown	P	4	-	-
**21b+**	27	Single	Primary	Business	Yes	Unknown	P	12	-	-
**22b+**	28	Mar	Primary	H/wife	Yes	Positive	P	12	-	-
**23b+**	29	Mar	Primary	Business	Yes	Unknown	P	12	-	-
**24b+**	28	Mar	Primary	Business	No	Unknown	P	1	-	-
**25b+**	24	Single	Primary	H/wife	Yes	Unknown	D	-	16	Neg
**26b+**	33	Mar	Primary	Business	Yes	Unknown	D	-	18	Neg
**27b+**	33	Mar	Primary	Business	Yes	Positive	P	4	-	-
**28b+**	30	Mar	Primary	H/wife	Yes	Positive	D	-	12	Neg
**29b+**	25	Cohab	Primary	H/wife	Yes	Positive	P	-	16	-
**30b+**	32	Cohab	Primary	H/wife	Yes	Unknown	D	-	32	Neg
**31b+**	21	Cohab	Primary	H/wife	Yes	Positive	D	-	12	Neg
**32b+**	25	Single	College	Nurse	No	Unknown	D	-	6	-
**33b+**	33	Mar	Primary	H/maid	Yes	Unknown	P	16	-	-
**34b+**	31	Mar	Non	H/wife	Yes	Negative	P	12	-	-
**35b+**	27	Single	Primary	H/maid	No	Unknown	D	-	12	-

ID no with a = Option A, with b = Option B, with b+ = option B also asked about B+.

Marital status: Mar = Married, Div  = divorced, Cohab = cohabiting.

Business  =  small-scale business.

The majority of the participants were in favor of Option B compared to Option A. Some of the women asked about B+ favored it as they agreed that they would eventually need ART for their own survival. Some were against B+ anticipating loss of motivation after protecting the child, fearing drug side-effects and not feeling ready to embark on lifelong medication. Some were undecided.

### Themes and categories

The findings are grouped into three main themes; three categories under the first theme, no categories under the second theme and three categories under the third theme. The first category in the first theme has two sub-categories.

### Theme I: Embracing Option B to minimize stigma and enhance ART adherence

The participants favored Option B (ART to mother with a CD4 count of >350 cells/µL during the breastfeeding period) over Option A (ARV prophylaxis to the infant during breastfeeding instead). The reasons for choosing Option B were grouped into three main categories: I) HIV-associated stigma, II) fear of drug side-effects and III) difficult logistics for postnatal drug adherence.

### Category I: HIV-associated stigma

It was very obvious during the interviews that there is still extensive HIV-associated stigma and discrimination in the community that forces HIV-infected individuals to desist from all practices that could reveal their HIV status. The unique PMTCT practices including ART adherence are now well known in African societies making it even more difficult for HIV-infected individuals to hide their status. Three sub-categories were identified under this category.

### Sub-category i: The perceived stigma of giving medicine to the child every day and abiding to optimal PMTCT guidelines

Most of the women explained how hard it was to give medicine to a healthy baby every day throughout the breastfeeding period. They stated that since people live in close proximity with family and neighbors, it is very easy to be noticed when doing something a bit unusual like giving a healthy baby medicine every day. Giving medicine is perceived as a sign of illness.

*“…when people around you see you giving medicine to your child every day, they keep asking why. It's difficult to lie every day. In the end, they will start gossiping behind your back.” ….. (30 years, married, completed primary education, employed)*

Another woman explained how such stigma might even affect the child later in life:

*“……so when they see the child taking medicine every day, they will point fingers at the child when it is very young and may discriminate against him when he goes from house to house…….. This will make the child very uncomfortable and he won't be free to play with other children.”(34 years, married, completed primary education, housewife)*

Today, practices and behaviors connected to pregnancy and delivery are closely observed in society as a way of finding out a woman's HIV status. This curiosity from neighbors and relatives makes it very difficult for an infected woman to adhere to clinical guidelines. Women expressed how they were striving to keep their own medication a secret, but that they found it even more difficult to conceal the fact that they were medicating their child;

*……. “People are curious to know why I'm taking medicines but I tell them that the medicine is for high blood pressure. So, if I give the child medicine every day, they will be surprised. What problem does the child have?! So, I better take medicine rather than the child…..” (28 years, single, completed primary education, small-scale businesswoman)*

### Sub-category ii: Breastfeeding stigma-related challenges

The aim of both Options A and B postnatally is to prevent HIV transmission through breast milk. The community perceives someone who does not breastfeed as likely to be HIV-infected. Therefore some women were happy that both options provided an opportunity to breastfeed for as long as they wished and still protect the baby while avoiding queries from the community.

*“……I would like a woman who is infected to breastfeed because you know the community; for example, if I give birth, then after giving birth if I do not breastfeed, and if the community that surrounds me sees that, they will see a problem. You see! But if you breastfeed for six months, or for a year, no one will query your health status; you take care of yourself...” (38 years, married, completed primary education, small-scale businesswoman)*

Another woman added:

*“What I hear from the neighbors is that an infected woman can transmit the virus to her child through breast milk, people in the neighborhood know that when a woman delivers and doesn't breast feed then she must be HIV infected.”(34 years, married, completed primary education, housewife)*

Nevertheless some women in both options seemed not to believe that postnatal ART can prevent transmission to the child so they would rather breastfeed for a short time (just to fool people around them) and stop as early as the first testing of the child's HIV status was negative.

*“…..when the first screening test is done, if you are lucky then you get a negative result. It's good if you continue protecting your child* [stop breast feeding] *because if you breastfeed and you know that a big percent of transmission occurs during breastfeeding then it's of no use to breastfeed..”. (34 years, married, primary education, housewife)*

### Category II: Fear of drug side-effects on the child and the perception that the child is too innocent to take ART

Some women were really concerned about drugs-related side-effects on their infant. They were also concerned about the long duration of medication throughout the entire breastfeeding period. They thought the mother should be more tolerable to take medicine than the child:

*……“I think I better take medicine as long as the child will get immunity through me because I don't know the strength of those medicines to the child….” (32 years, married, completed primary education, housewife)*

Another woman emphasized the importance of not giving medicine to the child:

*….. “I will choose to take medicine because you never know, maybe he* [child] *can experience side-effects in relation to my condition…..But I vomit… They get out, you see, after that I have no desire to take them again. How can you give such a thing to your child?”(29 married, completed primary education, small-scale businesswoman)*

Another woman feared future drug-related complications for the child:

*“….Just because I feel it is not good to give medicine to a small child; it may affect him in future. Because his body system is not matured…so I think it is better for the mother to take medicine instead of giving it to the child….” (27 years, married, college, agriculturalist)*

Several women were against the idea of giving daily drugs to their newborns because they perceived it as a sort of a punishment to an innocent child especially since the baby was healthy. HIV infection and its outcomes are still to a large extent regarded in Tanzanian society as a punishment for promiscuity.

*“Because it's an innocent creature, why give him (medicine)? The child doesn't know anything but yet has to take medicine …….. At least I understand that it's my fault and that the baby is innocent. If I were to choose, I would take the drugs and just let the baby breastfeed.”(34 years, married, completed primary education, housewife)*

### Category III: Challenging logistics for postnatal drug adherence

Women also said that they found it inconvenient to administer drugs to their infants. They felt that having to return back to their normal duties did not allow them to spend enough time with their child. This was especially difficult for employed women who only have 3 months maternity leave before they need to go back to work leaving the child under the care of other people in the household. Exclusive breastfeeding also then becomes very difficult to adhere to in reality especially for those who have not disclosed their HIV status. Most women argued that it is more convenient to take medicines themselves rather than giving them to the child.

*“…….Sometimes you may have to leave the child; who will give the medicine to the child if you do not want people to know? They will be surprised: why should this child take medicine every day? Isn't it better for you take it yourself? (27 years, married, college, agriculturalist)*

Some women found that daily routines were not helping them to maintain confidentiality:

*“I can't stay with the baby every day. Sometimes emergencies happen and may need to be away for long periods and it may be time to take drug and you are not there. So I think it's much better I take the drugs myself because I will know when and how to take them.”(34 years, married, completed primary education, housewife)*

In addition, the women also explained how challenging it was to give medicine to young children every day as most of the children do not like taking any type of medicine. Their perception is that the taste of the medicine is usually unpleasant and it may be difficult to make the child take it on time and at the right dose throughout the breastfeeding period as they may spit or vomit it out.

*……..“The action of giving the child medicine is challenging as sometimes the child is crying and you don't know whether the medicine is bitter or not but you just have to give it..… (21 years, married, completed primary education, housewife)”*

### Theme II: Eagerness to prolong life through Option B+

Some of the participants who were also asked for their views on Option B+ were very positive towards the idea of taking drugs for life after being diagnosed with HIV during pregnancy. They all gave the same reason that this will help them have a better and longer life enabling them to raise their children for a much longer time before they die.

*“……… it's better to continue with them [ART] throughout because they protect you from minor problems like falling sick frequently; getting rashes….. Yes, you should continue; you will eventually get used to them [medicine]! (28 years old, completed secondary education, married, small-scale businesswoman)*

*“I will take medicine for life and I will adhere to all instructions they give and I will take them all the time to protect myself so that I live longer and take care my children. (33 years, married, completed primary education, small-scale businesswoman)*

One woman was of the opinion that if people are well informed of the benefits of ART, they may adhere well to treatment.

*…..“As long as they know that if you take medicine you prolong your life compared to if you don't, so, which one is better? It's better to take medicine for life!.......... They will accept because they know if you take medicine you may die next year but if you don't take them you may die this year; so, you better take them! No one will say ‘no, let me just die’ and stop taking medicine.” (27 years, married, completed primary education, small-scale businesswoman)*

### Theme III: Feared obstacles to Option B+ adherence

On the contrary, some other participants who were also asked for their views on Option B+ expressed negative opinion towards Option B+ since they thought they would lose motivation after knowing they had protected the child, feared drug side-effects and were uncertain that they would be able to overcome the challenges of lifelong daily medication.

### Category I: Loss of motivation to continue with ART after protecting the child

Some women clearly expressed that they were persevering with the whole process of adhering to medication and breastfeeding regulations just for the sake of the child. One of them said:

*…… “I'm mature enough, I can tolerate taking medicine during this period just to make sure that the child is protected.” (28 years, single completed primary education, small-scale businesswoman)*

…..“ *Most of them* [women] *won't live to reach old age. Taking medicine every day is challenging so you may find that once a person is not pregnant she will obviously stop taking medicine unless she falls sick, at which point she will start taking them again. (29 years, married, completed primary education, small-scale businesswoman)*

### Category II: Fear of experienced drug side-effects

Women explained their concerns about the side-effects they experienced when put on ART as a barrier for lifelong medication. They also pointed out clearly how the side-effects reduced their motivation to adhere to the prescribed drug treatment. Since most pregnant women were put on ART prophylactically and they had not experienced any HIV-related symptoms, they questioned if they would have enough motivation to continue with the ART after knowing they had protected the child.

*“I've tried to take and get used to the night dose but duh….I'm not good at all after the night dose but I tried to take them but I can't manage…. It's disturbing a lot!!! Sometimes you become weak, you have problems sleeping and in the head it feels as if there is fire… (28 years, single, completed primary education, small-scale businesswoman)*

They associated prolonged use of ART with their previous experience:

*…..“Taking medicine for life time! It is good advice but I can see most people will not take them because it is tiresome. There are other medicines you take in the first week that cause dizziness so other people will end up saying ‘I can't’… (29 years, married, completed primary education, small-scale businesswoman)*

### Category III: The challenges of lifelong daily medication

Women expressed how difficult it is to be on chronic medication. Adherence appeared challenging as it seemed very difficult to incorporate taking drugs into their daily schedule, partly due to the fact that most do not feel ill, have busy and demanding lives and given the fact that the drugs are associated with the stigma of HIV.

…..“*The challenge is not only taking the same medicine but also taking it every day... At least it should be once per month or week….. But you have to take it every day! It's very tiring… Sometimes I forget to take them at the right time and other medicines I tried to take them but I can't. (41 years, married, completed primary education, small-scale businesswoman)*

*……..“There is the issue of forgetting to take medicine and you are told not to skip even a single day so what will happen?... It is possible* [to forget] *because I may eat and I may fall asleep without taking them… for example the family planning pills if you use them you may forget to take them so, you notice that it is better to use injection……“I will advise them to use alternative ways because they are still looking for medicine for treating HIV and have not succeeded so far. So, they have to look for injection so that people will not forget like family planning.”…… (28 years, married, completed primary education, housewife)*

The participants expressed their past experiences with ART adherence in the community. They noted that when people get tired of taking the medicines, they simply throw them away but keep telling their health care providers that they are still adhering to their drug regimen:

*…. “if you tell that person to take medicine every day… will not use them instead they throw them away under the bed. There are so many people doing that. Taking medicine every day is confusing people. Whenever they come to the clinic, they will make sure they take medicine and the next visit you do the same but I know when you give us them to take home, others do not take them. (33 years, married, completed primary education, small-scale businesswoman)*

## Discussion

WHO has advised each country to explore the acceptability of the new PMTCT options (A, B, and B+) before implementation, as this is a critical indicator of how well the intervention will work [23]–[25]. Such information is critical to cover the gap between what people think and feel in their local context with regard to adherence and retention issues versus what the government/various programs expect them to do. It is also part of human rights to let people choose what is best for them as both interventions have the same efficacy [23].

Our study reveals that the majority of the interviewed women preferred Option B for PMTCT; the main reason being the fear of stigma and discrimination associated with HIV. It is disappointing and surprising to find that the degree of stigma remains so strong, over three decades into the HIV epidemic in Tanzania. HIV/AIDS is still thought of as a punishment for sinning and sexual promiscuity [33]. People living with HIV and even more so, those on ART, are often considered a threat to society and “dead-to-be” individuals who may intentionally transmit the virus to others [34]. The impact of stigma manifests itself both at individual and community level [35], [36] and has been a major obstacle to the effective large-scale implementation of and adherence to PMTCT and ART [37]. It is very clear from this study that the majority of pregnant women living with HIV fear and experience both individual- and community-level stigma that clearly interferes with effective implementation of PMTCT and lifelong ART. The implication of this observation is very important as the country moves towards implementation of PMTCT Option B+.

Stigma makes it very difficult to adhere to treatment especially if there is no HIV status disclosure at family level. In such cases it becomes much easier if the woman swallows the drugs herself compared to giving them to the child everyday throughout the breastfeeding period. Giving daily medicine to children can also be tricky especially as they grow and when the mother has to work or travel to earn a living, as is the case for most of the women in this study. Children can easily vomit or spit out medicine, making the dosage very questionable. It is also very easy to forget to give medicine at the right time especially when the child is not sick and is left at home with other carers which is usually the case in this setting. Since HIV infection is considered as a punishment for sin, the majority of women consider taking ART as a punishment for something they think that their innocent newborns do not deserve to go through.

Most of the women were very concerned about the side-effects of ART and were more worried that it would cause suffering to the “innocent” newborn who cannot express its feelings verbally. Women expressed how they modified their doses and decided which part of the ART combination regimen to take and which to stop taking, indicating poor adherence that may also result in drug resistance, a more complicated and expensive issue to handle in a poor-resource setting like Tanzania. Such practices are a major drawback when Option B+ is considered including the initiation of ART for life during pregnancy.

We noted that exclusive breastfeeding is also very challenging as most women in this poor-resource context do not have the luxury of staying at home with the baby throughout the breastfeeding period. Most women would have to return to their normal duties three months after their baby is born. Again exclusive breastfeeding is not a normal practice in this society and today also known to be strongly associated with HIV, making it stigmatizing. Nevertheless women with a better income and who are less stigmatized find it easier not to breastfeed at all or to breastfeed for a short period until the first HIV PCR test of the child is negative. Early cessation of breastfeeding is less stigmatizing because the mother does not have to worry about taking and adhering to the drugs for a long time. At the same time the child gets colostrums, which is the best milk, and those around the woman will know that she has breastfed, removing their doubts that she is HIV-infected.

This study also found that only a minority of the women were in favor of Option B+, i.e. lifelong ART post-delivery regardless of CD4 count. They were of the opinion that they needed to prolong their lives so that they could care for their young ones. On the contrary, women who were against B+ painted a picture of questionable drug adherence as we learned that their main incentive to stay on ART was to protect the child while hiding their HIV-status. This finding is similar to what we found in the Mitra Plus study, where women eligible for ART for life were followed up for 24 months postnatally and it was noted that the majority had detectable viremia suggesting poor adherence despite having a CD4 count <200 cells/µL [32]. In-depth interviews with the Mitra Plus mothers revealed that the major reason for poor adherence was that they lost motivation to take drugs once they had discovered that they had protected the child from becoming infected [32]. Taking drugs everyday increases the risk of eventually revealing one's HIV status, in turn making life much more difficult and putting one in a dangerous social position in today's Tanzanian society.

The reasons and circumstances that pushed Malawi to adopt Option B+ [25] are the same for Tanzania, i.e. high fertility rate, lack of access to CD4 cell count analysis, shortage of staff (both lab, clinic and inpatient) to implement the complicated drug regimen for option A and B, and being able to perform proper follow-up procedures. The theoretical benefits acquired through Option B+ in resource-limited settings are many and include: simpler instructions at clinic level i.e. the “one size fits all approach”; protection of future pregnancies already from conception; a strong reduction in the risk of sexual HIV transmission to a sero-negative partner; and lower risk of late re-initiation of ART when the woman really needs it for her own health. A cross-sectional analysis done at a rural referral hospital in Tanzania and assessing uptake of PMTCT recommendations identified gaps at all steps of the PMTCT implementation pathway [38]. The authors recommended B+ for Tanzania arguing that it potentially could cover most of these gaps, increase PMTCT coverage and contribute to the goal of eliminating MTCT in sub-Saharan Africa by 2015.

Nevertheless, several parties are concerned about program feasibility and acceptability, medical safety and benefits, ethical issues and economic consequences of implementation of Option B+ [39]. Preliminary data from a nation-wide cohort in Malawi, the first country to introduce Option B+ at national level, shows that 17% of Option B+ clients were lost to follow-up within 6 months. Furthermore, Option B+ clients who started ART during pregnancy were five times more likely to drop out after their initial visit compared to women who started ART due to a low CD4 count and/or were in WHO clinical stage 3 or 4 [40].

Other concerns raised are that it may be difficult to justify the favoring of newly delivered mothers with young children over men and non-pregnant women or women with older children, particularly in view of the fact that there is no data to suggest that newly delivered women have a higher involvement in discordant relationships than other women or men in society [39]. The major challenges are to sustain and afford the necessary levels of drug procurement and distribution in weak health systems, and to achieve and maintain adherence and retention of women in treatment programs [41]–[43]. Our experience from a previous study [32] of poor ART adherence postnatally in women treated for their own health shows that it is very important to have repeated laboratory monitoring with viral load testing for early detection of treatment failure and to combat the risk of drug resistance development.

### Limitations

As with most qualitative study results, the findings of this study cannot be generalized beyond the context we studied, but can be transferred to similar contexts. Dar es Salaam is an urban center where women are presumably more aware of HIV-related issues and living conditions compared to women living in rural areas. Given the rapid urbanization of Africa, causing the majority of Africans to live in urban rather than rural areas, women in rural areas may have different views. Although the majority (98%) of pregnant women in Tanzania use RCH clinic, the results of these interviews should not be generalized to pregnant and HIV-infected women who do not attend RCH clinics. It would also be interesting to obtain the views of men but it is almost impossible to interview HIV-infected male partners of pregnant or newly delivered women due to stigma. The majority of women disclosed their HIV status to their partners but most of their partners were not ready to be tested or disclose their status. Since Option B+ implementation has just started in Tanzania, the women did not have any experience of their own of being on life-long ART. However, the majority had experience of taking ART during pregnancy and breastfeeding and had felt the stigma associated with HIV infection and ART. We also noted that some women were telling us what they knew we wanted to hear (social desirability bias), but given that the majority acknowledged adherence difficulties and lack of incentives to stay on ART and the high consistency in their reports, we think that the validity of the information collected is high.

### Conclusion

This qualitative study reveals that the majority of women prefer Option B for PMTCT during the breastfeeding period mainly because of the stigma and discrimination associated with HIV/AIDS and ART in the society, fear of side effects experienced when one is on drugs and the difficult logistics for drug adherence postnatally. Women's views on Option B+ were divided. Those in favor of B+ were highly determined to prolong life for their own sake and were concerned for the welfare of their children. The others questioned their own ability to adhere to life-long ART thinking that they would be less motivated to take drugs after protecting their child, and fearing the drug side effects and the challenges of chronic daily medication. Since Tanzania has recently adopted Option B+, women with a CD4 count of >350 cells/µL should be counseled about the possibility to “opt-out” from ART after cessation of breastfeeding. Repeated counseling focused on drug adherence and its value for asymptomatic women even when not pregnant or breastfeeding is of great importance. It is also very important to introduce monitoring and evaluation of the program which includes laboratory-based virological and immunological assessment of a randomly selected cohort on Option B+.
